# Structural, Magnetic and Vibrational Properties of Van Der Waals Ferromagnet CrBr_3_ at High Pressure

**DOI:** 10.3390/ma16010454

**Published:** 2023-01-03

**Authors:** Olga Lis, Denis Kozlenko, Sergey Kichanov, Evgenii Lukin, Ivan Zel, Boris Savenko

**Affiliations:** 1 Frank Laboratory of Neutron Physics, Joint Institute for Nuclear Research, 141980 Dubna, Russia; 2 Department of Nuclear-Physical Materials Science, Institute of Physics, Kazan (Volga Region) Federal University, 420008 Kazan, Russia

**Keywords:** neutron diffraction, high pressure, crystal and magnetic structure, phase transition, van der Waals ferromagnet

## Abstract

The crystal and magnetic structures of van der Waals layered ferromagnet Cr${\mathrm{Br}}_{3}$ were studied using X-ray powder diffraction and neutron powder diffraction at pressures up to 23 GPa at ambient temperature and up to 2.8 GPa in the temperature range 6–300 K, respectively. The vibration spectra of Cr${\mathrm{Br}}_{3}$ were studied using Raman spectroscopy at pressures up to 23 GPa at ambient temperature. The anomalous pressure behavior of structural parameters and vibrational modes was observed, associated with a gradual isostructural phase transition in the pressure range 2.5–7 GPa. The Curie temperature *T_C_* reduced rapidly with a pressure coefficient $dT_{C}/dP=-4.1\left(4\right)$ K/GPa. A full suppression of the ferromagnetic state was expected at *P_C_*~8.4 GPa, where onset of the antiferromagnetic spin arrangement or magnetically disordered state may take place. Anomalies in Raman spectra at *P*~15 GPa point to another possible phase transformation in Cr${\mathrm{Br}}_{3}$, which may be related to the proximity of metallization of this van der Waals ferromagnet.

## 1. Introduction

A discovery of intrinsic ferromagnetism in two-dimensional (2D) atomically thin van der Waals (vdW) magnets initiated comprehensive fundamental and applied research of these materials, demonstrating challenging physical phenomena and highly promising for applications in spintronics and electronics [1,2,3,4,5,6,7,8]. The layered structure and the vdW gap between the layers in two-dimensional vdW magnets provide different routes to manipulate their magnetic properties. In particular, an application of high pressure through changing interatomic distances and angles gives an effective way to adjust both magnetic and electronic properties without changing the chemical composition [9,10]. Applying pressure predominantly compresses the weakly bonded interplanar distances and switches the lattice toward a 3D character in a van der Waals magnet. As an example, pressure causes changes in the stacking magnetic order of layers, which induces a phase transition from antiferromagnetic to ferromagnetic in the bilayer system CrI_3_ [9,10,11].

Recent studies have demonstrated a rich variety of pressure-induced phenomena in vdW materials. In antiferromagnet FePX_3_ (X = S, Se) pressure-induced insulator–metal transition, structural transitions, and spin crossover, and also the emergence of superconductivity (in FePSe_3_), were reported [12,13,14]. In FePS_3_, the initial antiferromagnetic interplanar coupling with strong 2D character is switched by high pressure to a ferromagnetic interplanar one and the magnetic state evolves from 2D-like to 3D-like character [15]. In CrSiTe_3_, the ferromagnetically ordered state transfers to the paramagnetic one with increasing pressure, accompanied by a structural transition and the appearance of superconductivity [16]. In Cr_2_Ge_2_Te_6_, pressure-induced spin reorientation was discovered, which drove equilibrium magnetization from the c axis to the ab plane [17]. The vdW ferromagnets demonstrate a different response of *T_C_* under pressure. While it increases in CrI_3_ and VI_3_ [18,19], a suppression of *T_C_* was found in Cr_2_Ge_2_Te_6_ [20], CrBr_3_ [21], Fe_3_GeTe_2_ [22]. A pressure-induced semiconductor-to-metal transition was observed in CrI_3_, accompanied by strengthening of the AFM interactions [23]. The isostructural phase transition in CrCl_3_ was detected at about ~10 GPa, followed by electronic topological transition and metallization at P~30 GPa [24,25].

Among the vdW CrY_3_ family (Y = Cl, Br, I), the structural and magnetic properties of the CrBr_3_ representative remain weakly explored. Recently this material has attracted particular attention due to pronounced negative thermal expansion effects [26,27,28]. It crystallizes in a rhombohedral structure of R$\bar{3}$ symmetry, where the Cr atoms form a honeycomb lattice, which is clenched by two atomic planes of Br atoms. The ferromagnetic order is settled in CrBr_3_ below the critical temperature of 36 K. In this work, the structural, magnetic and vibrational properties of CrBr_3_ under high pressure were studied by a combination of X-ray, neutron diffraction and Raman spectroscopy techniques.

## 2. Materials and Methods

The polycrystalline CrBr_3_ sample was made from single crystals supplied by HQ Graphene. The X-ray powder diffraction (XRD) experiments at high pressures up to 23 GPa were performed using the SAXS/WAXS Xeuss 3.0 system (XENOCS SAS, Grenoble, France) with the Dectris Eiger 2R 1 M detector, Mo $K_{\alpha }$ radiation (λ = 0.71078 Å). The Almax Plate type diamond anvil cell was used. Diamonds with culets of 250 µm were taken. The sample was loaded into a hole of 150 µm diameter made in the Re gasket to about 20 µm thickness.

Neutron powder diffraction (ND) measurements were performed in the temperature range 6–300 K at pressures up to ~3 GPa using a DN-6 diffractometer [29] at IBR-2 high flux pulsed reactor (FLNP JINR, Dubna, Russia). A powder sample volume of about 2 mm^2^ was loaded into the sapphire anvil high pressure cells [30]. The spherical holes with a diameter of 2 mm were drilled at the anvil’s culets for the quasi-hydrostatic pressure distribution at the sample surface. Several tiny ruby chips were placed at different points of the sample surface and the pressure was determined by a standard ruby fluorescence technique. Measurements of the pressure distribution on the sample yielded typical pressure inhomogeneities of ±15%. The diffraction patterns were collected at the scattering angle of 90° with a resolution of Δd/d = 0.022. The neutron and X-ray diffraction data were analyzed by the Rietveld method using the Fullprof 7.6 software [31].

Raman spectra at ambient temperature and pressures up to ~20 GPa were collected using a LabRAM HR spectrometer (Horiba Gr, Montpellier, France) with a wavelength excitation of 633 nm emitted from He–Ne laser, 1800 grating, a confocal hole of 100 μm, and ×20 objective. The Almax Plate type diamond anvil cell was used.

## 3. Results

### 3.1. X-ray Diffraction

**Figure 1:**
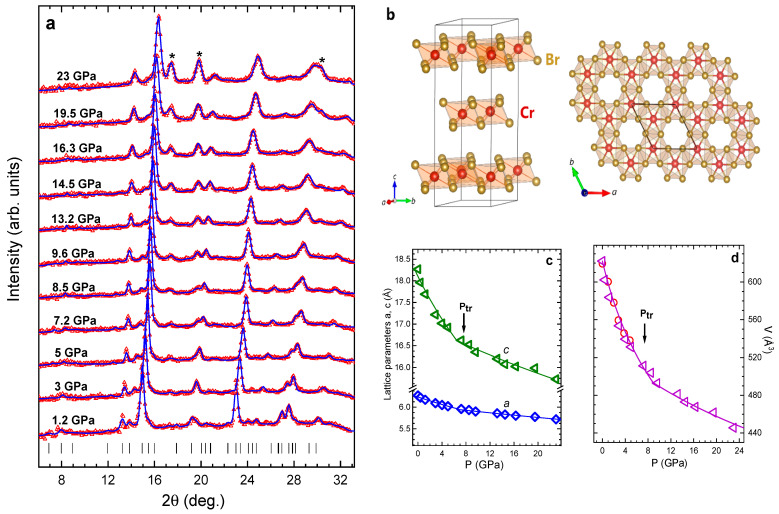
(**a**) X-ray diffraction patterns of CrBr_3_ obtained at selected pressures and ambient temperature, and refined by the Rietveld method. Experimental points and calculated profiles are shown. The tick below marks the calculated positions of the structural peaks of phase of rhombohedral phase of CrBr_3_. The asterisks (*) indicate additional diffraction peaks from the rhenium gasket; (**b**) Schematically representation of rhombohedral crystal structure of CrBr_3_. The unit cell (**left**) and top view (**right**) are shown. The orientation of the crystallography axes is presented; (**c**) The lattice parameters of CrBr_3_ as a function of pressure. The phase transition pressure *P_tr_* is labeled. The solid lines are linear fit of experimental data; (**d**) The pressure dependence of unit cell volume of the ambient and high-pressure phases of CrBr_3_, fitted by the third order Birch-Murnaghan equation of state (1). The red circles represent the obtained values for unit-cell volume from ND data (see below).

The XRD patterns of CrBr_3_ measured at selected pressures and ambient temperature are shown in Figure 1a. They correspond to the initial rhombohedral crystal structure of R$\bar{3}$ symmetry. The values of lattice parameters determined at ambient conditions, *a* = 6.270(4) and *c* = 18.269(5) Å, were consistent with those obtained previously [6,26,32]. At moderate pressures, the lattice compression of CrBr_3_ was markedly anisotropic (Figure 1c) with the *c* lattice parameter nearly twice as compressible as the *a* parameter. The corresponding average compressibility values [$k_{i}=-(1/a_{i0}){\left(da_{i}/dP\right)}_{T}$] were $k_{a1}$ = 0.0067(3) and $k_{c1}$ = 0.0147(2) GPa^−1^. At transition pressure *P_tr_*~7 GPa, a significant change in pressure behavior of the *c* lattice parameter occurred (Figure 1c). Its compressibility was reduced by about four times to $k_{c2}$ = 0.0035(4) GPa^−1^, becoming comparable with that of the *a* parameter, which decreased by more than twice to $k_{a2}$ = 0.0026(1) GPa^−1^. In the absence of qualitative changes in the XRD patterns, one may suggest that the observed lattice compression anomaly was associated with the isostructural phase transition into a denser phase with reduced distance between the vdW layers. A somewhat similar phenomenon was also observed in a related compound CrCl_3_ at ~11 GPa [25]. It should be noted that the pressure-induced phase transition in CrY_3_ materials occurred in a smoother manner in comparison with FePX_3_ (X = S, Se) systems, where first-order structural phase transitions at ~13 GPa and ~8 GPa, respectively, were accompanied by discontinuous drops of the lattice parameters [12,14]. This observed large-volume collapse has been proposed to be associated with spin-crossover and insulator-metal transitions. It is also interesting to note that, in contrast to CrY_3_, in ternary CrSiTe_3_ and Cr_2_Ge_2_Te_6_ systems, a pressure-induced suppression of the crystalline phase, followed by an amorphization process, was reported [33,34].

The volume compressibility data of CrBr_3_ (Figure 1d) were fitted by the third-order Birch–Murnaghan equation of state (1) [35]:

(1) $$P=\frac{3}{2}B_{0}\left(x^{-7/3}-x^{-5/3}\right)\left[1+\frac{3}{4}\left(B^{\prime }-4\right)\left(x^{-2/3}-1\right)\right]$$

where *x* = $V/V_{0}$ is the relative volume change; $V_{0}$ is the unit cell volume at ambient pressure; $B_{0}$, $B^{\prime }$ are the bulk modulus [$B_{0}=-V{\left(dP/dV\right)}_{T}]$ and its pressure derivative [$B^{\prime }={\left(dB_{0}/dP\right)}_{T}]$.

The obtained bulk moduli for the ambient pressure and high-pressure phases were $B_{0}=23\left(4\right)$ and $B_{0}=94\left(3\right)$ GPa at fixed $B^{\prime }=4$. The ambient-pressure phase value was comparable with those obtained for CrCl_3_ $(B_{0}=28\left(2\right)$ GPa) [25]; CrSiTe_3_ ($B_{0}\sim 37\left(1\right)$ GPa, $B^{\prime }=3.82$) [34]; and Cr_2_Ge_2_Te_6_ ($B_{0}=39.2$ GPa, $B^{\prime }=3.8$) [36].

### 3.2. Neutron Diffraction

**Figure 2:**
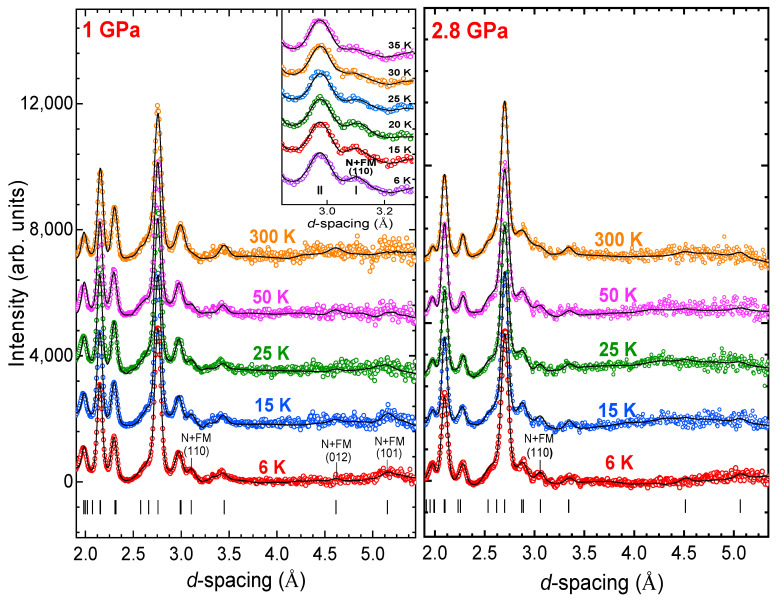
Neutron diffraction patterns of CrBr_3_ measured at selected pressures and temperatures and refined by the Rietveld method. The experimental points and calculated profiles are shown. Ticks below represent calculated positions of the nuclear peaks of the rhombohedral phase of CrBr_3_. The magnetic contribution into nuclear peaks is labeled as “N+FM”. Inset: the pressure evolution of the characteristic peak with additional magnetic contribution.

**Figure 3:**
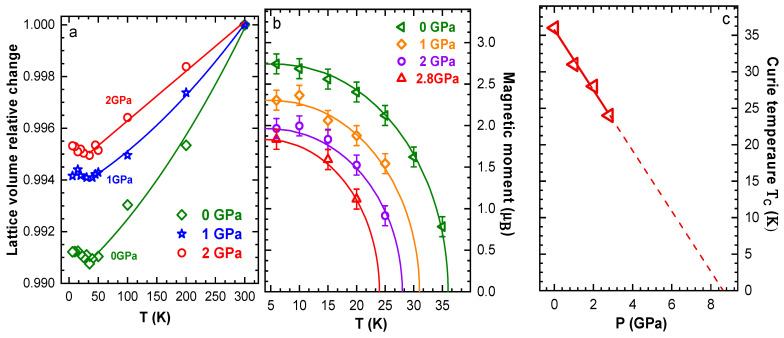
(**a**) The temperature dependences of the unit-cell volume obtained from neutron diffraction and normalized to the ambient temperature values; (**b**) The temperature dependences of the ordered Cr^3+^ magnetic moments at selected pressures. The solid lines represent fitting by function described by Equation (2); (**c**) Curie temperature as a function of pressure and its linear extrapolation.

Neutron diffraction patterns of CrBr_3_, measured at selected pressures and temperatures, are shown in Figure 2. At ambient pressure below *T_C_* = 36(2) K, the appearance of additional magnetic contribution to intensities of the peaks (110), (012), (101) was revealed, indicating formation of the FM state [26]. The ordered magnetic moments were oriented along the *c*-axis and their values were *M* = 2.74(8) µ_B_. Remarkably, the strongly anisotropic thermal expansion of the CrBr_3_ lattice with the pronounced variation of the *c* lattice parameter was also observed, even upon compression up to 2.8 GPa. As a result, the negative thermal expansion of the unit cell volume for *T* < *T_C_* was preserved in the studied pressure range (Figure 3a). This effect was associated with a strong spin–phonon coupling [26]. The average volume thermal expansion coefficients ($a_{V}=\left(1/V\right)(dV/dT))$ were $a_{V}=2.2\times 10^{-5}{\;K}^{-1}$ for *T* > *T_C_* and $a_{V}=-1.8\times 10^{-5}K^{-1}$ for *T* < *T_C_* at *P* = 1 GPa and $a_{V}=1.9\times 10^{-5}{\;K}^{-1}$ for *T* > *T_C_* and $a_{V}=-1.4\cdot 10^{-5}K^{-1}$ for *T* < *T_C_* at *P*= 2 GPa. The obtained coefficients for regions below *T_C_* decreased with application of pressure, and this effect correlated with a suppression of long-range magnetic ordering, as we discuss below.

With a pressure increase, a progressive reduction of the magnetic contribution, to the (110) and (101) peaks, was detected (Figure 2), evidencing reduction of magnetic moment and suppression of magnetic ordering.

The temperature dependences of the Cr^3+^ magnetic moments at selected pressures are shown in Figure 3b. They were analyzed in the framework of the molecular field model using expression:

(2) $$\frac{M}{M_{0}}=B_{s}\left(\frac{3S}{S+1}\frac{M}{M_{0}}\frac{T_{C}}{T}\right)$$

where $B_{s}$ is the Brillouin function, S is the spin of the system (S = 3/2) and $M_{0}$ is the ordered magnetic moment at T=0. The obtained Curie temperature decreased under pressure with a coefficient $dT_{C}/dP=-4.1\left(4\right)$ K/GPa (Figure 3c). Simultaneously, the ordered Cr^3+^ magnetic moments of the FM state at *T* = 6 K decreased from 2.74(8) μ_B_ to 1.8(4) μ_B_ in the pressure range 0–2.8 GPa. These decreasing trends were also detected for this compound earlier from magnetization measurements in a more restricted pressure range up to 1 GPa, providing a comparable value of $dT_{C}/dP=-3.1$ K/GPa [32]. The pressure behavior of CrBr_3_ was opposite to CrI_3_, where a pronounced increase of *T_C_*, by about 7.5%, for an applied pressure of 1 GPa occurred [18].

The extrapolation of the experimental Curie temperature pressure dependence of our work showed (Figure 3c) that the *T_C_* value turned to zero at *P_C_*~8.4 GPa. Therefore, one might expect a modification of the ground state in CrBr_3_ from FM to either AFM or a magnetically disordered state in the high-pressure phase for *P* > *P_C_*. The pressure evolution of magnetic exchange coupling constants in CrBr_3_ was theoretically studied by means of *ab-initio* calculations [37]. It was revealed that in a wide pressure range, the in-plane exchange constant $J_{in}$ for CrBr_3_ took a dominant value compared to the out-of-plane $J_{out}$, and it decreased rapidly. In contrast, a weak growth of $J_{out}$ occurred under pressure. The pronounced pressure-induced reduction of the $J_{in}$ exchange constant was a possible mechanism for a destabilization of the FM ground state in CrBr_3_. It also followed an evolution trend from the FM to AFM state upon the “chemical” pressure effect in the CrY_3_ family by halide ionic radius (and relevant lattice volume) reduction from I to Cl.

### 3.3. Raman Spectroscopy

**Figure 4:**
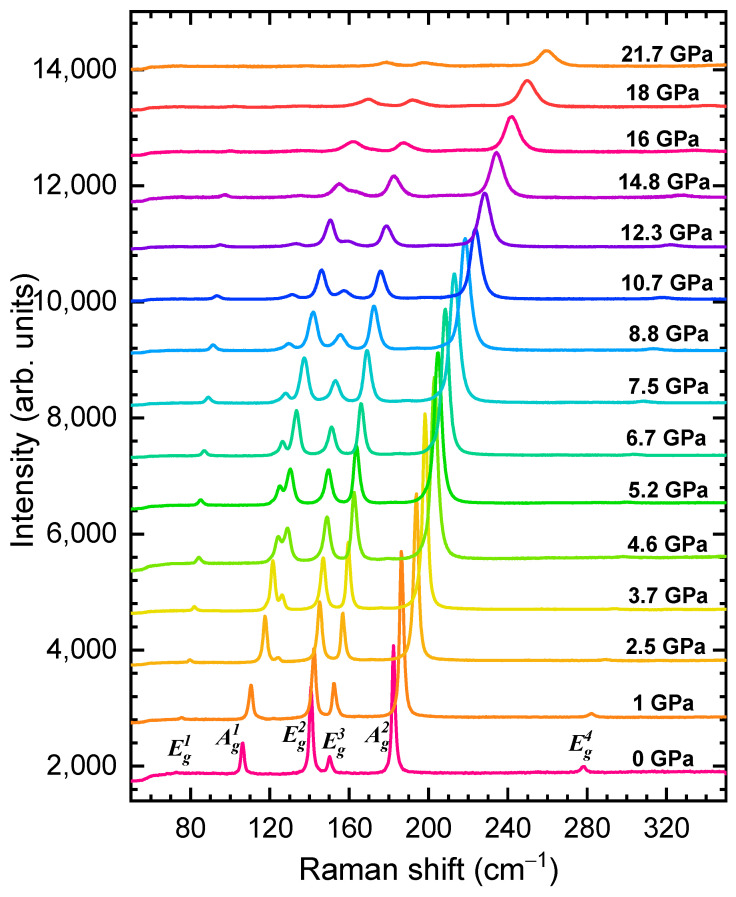
The Raman spectra of CrBr_3_ measured at selected pressures and room temperature.

The Raman spectra of CrBr_3_ measured at various pressures up to ~22 GPa are shown in Figure 4. At pressures below 2.5 GPa, six Raman active modes were observed, in consistence with the rhombohedral R$\bar{3}$ lattice symmetry [26,38,39,40]. They were assigned as 73.4 cm^−1^ ($E_{g}^{1}$), 106 cm^−1^ ($A_{g}^{1}$), 141.2 cm^−1^ ($E_{g}^{2}$), 149.6 (cm^−1^ $E_{g}^{3}$), 184.8 cm^−1^ ($A_{g}^{2}$), 279.5 cm^−1^ ($E_{g}^{4}$) (Table 1). According to [40], the $A_{g}^{1}$ and $A_{g}^{2}$ modes were mainly associated with in-plane and out-of-plane vibrations of Br atoms, respectively. It was demonstrated that the $E_{g}^{3}$ and $E_{g}^{2}$ modes were degenerate, resembling the in-plane shear motion of the Br atomic planes and also involving Cr atoms with out-of-plane displacements [40]. The pressure coefficients and calculated Gruneisen parameters γ for all the observed Raman modes are listed in Table 1.

At pressures above 2.5 GPa, the emergence of a new Raman mode was observed at 116.08 cm^−1^, further labeled as M_1_. Upon further compression, intensities of this mode and the $E_{g}^{3}$ one increased strongly, while the intensity of the $A_{g}^{1}$ mode rapidly suppressed (Figure 4). At *P* ~15 GPa, the $E_{g}^{2}$ mode merged with M_1_. Above this pressure, noticeable broadening of the Raman lines was found.

**Figure 5:**
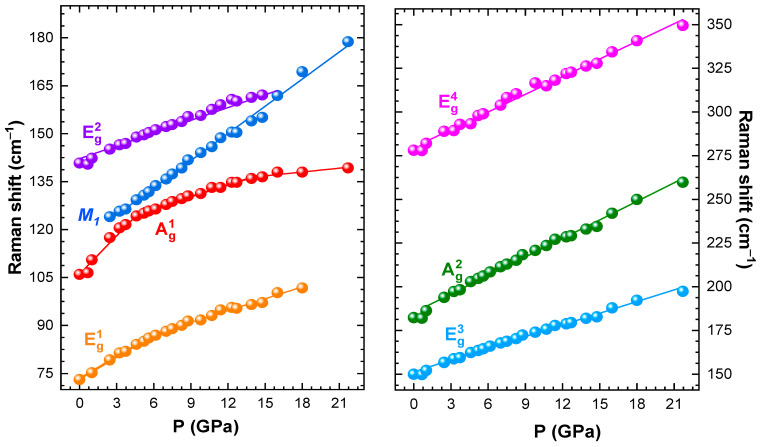
Pressure dependences of selected Raman shift for CrBr_3_. Solid lines are linear fits to the observed frequencies.

Most of the observed Raman mode frequencies, except for $A_{g}^{1}$ and $E_{g}^{1}$, increased nearly linearly under pressure (Figure 5). The pressure dependence of the $A_{g}^{1}$ mode demonstrated a sharp change of the pressure coefficient at *P*~6 GPa, correlating with the anomaly in the pressure behavior of the *c* lattice parameter (Figure 2c). A similar less pronounced change also occurred for the $E_{g}^{1}$ mode. These effects, along with observation of the M_1_ mode, were associated with the gradual isostructural phase transition, which likely started at *P*~2.5 GPa and evolved up to 7 GPa, becoming first visible in the Raman spectra and, subsequently, in the XRD patterns, when sufficient volume of the material was being transformed. The merging of the $E_{g}^{2}$ and M_1_ modes, along with another weaker modification of the $A_{g}^{1}$ mode pressure coefficient at 15 GPa (Figure 5), might be a signature of another phase transition. Taking into account the absence of qualitative changes in the XRD patterns, this transformation might be of an electronic nature, like the semiconductor to metal transition observed in CrI_3_ at pressures above 22 GPa [23] and in CrCl_3_ at ~30 GPa [24].

## 4. Conclusions

Our results demonstrated that the pressure-induced isostructural phase transition evolved gradually in vdW ferromagnet CrBr_3_ over a pressure range of 2.5–7 GPa. This transition emerged in Raman spectra around *P*~2.5 GPa by the appearance of an extra Raman mode and manifested finally in anomalies in the pressure behavior of the lattice parameters, unit cell volume, and $A_{g}^{1}$ and $E_{g}^{1}$ vibrational mode frequencies at *P*~6–7 GPa, when a volume of the pressure-induced phase became sufficiently dominant. The Curie temperature of CrBr_3_ reduced rapidly with a pressure coefficient $dT_{C}/dP=-4.1\left(4\right)$ K/GPa, implying instability of the initial FM order. A full suppression of the FM state and a magnetic transition to either AFM state or into a magnetically disordered one was expected at *P*~8.4 GPa. Additional anomalies in pressure behavior of Raman mode frequencies, detected at *P*~15 GPa, pointed to another phase transformation, presumably associated with the metallization process.

## Figures and Tables

**Table 1:** The assignment and frequencies of Raman mode, the pressure coefficients and mode Gruneisen parameters $\gamma_{i}$ for vibration modes of CrBr_3_ calculated for different pressure ranges. The mode Gruneisen parameters $\gamma_{i}$ are determined as $\gamma_{i}=B_{0}/v_{i}{\left(dv_{i}/dP\right)}_{T}$, where $B_{0}$ is the bulk modulus ($B_{0}=23\left(4\right)$ GPa for *P* < 6 GPa and $B_{0}=94\left(4\right)$ GPa for *P* > 6 GPa).

Mode Symmetry	*ν*_0_ (cm^−1^)	$dv_{i}/dP\;$ (cm^−1^/GPa)	$\gamma_{i}$
$E_{g}^{1}$	73.4	2.27 (*P* < 6 GPa) 1.23 (*P* > 6 GPa)	0.71 (*P* < 6 GPa) 1.45 (*P* > 6 GPa)
$A_{g}^{1}$	106.05	4.0 (*P* < 6 GPa) 1.22 (*P* > 6 GPa)	0.87 (*P* < 6 GPa) 0.96 (*P* > 6 GPa)
*M*_1_	116.08	2.82	2.29
$E_{g}^{2}$	142.18	1.33	0.22
$E_{g}^{3}$	149.6	2.64	0.4
$A_{g}^{2}$	184.8	3.57	0.44
$E_{g}^{4}$	279.56	3.39	0.27

## Data Availability

Not applicable.
